# AusTrakka: Fast-tracking nationalized genomics surveillance in response to the COVID-19 pandemic

**DOI:** 10.1038/s41467-022-28529-9

**Published:** 2022-02-14

**Authors:** Tuyet Hoang, Anders Gonçalves da Silva, Amy V. Jennison, Deborah A. Williamson, Benjamin P. Howden, Torsten Seemann

**Affiliations:** 1 Microbiological Diagnostic Unit Public Health Laboratory, The Department of Microbiology and Immunology, The University of Melbourne at The Peter Doherty Institute for Infection and Immunity, Melbourne, VIC Australia; 2 Public Health Microbiology, Queensland Public Health and Infectious Diseases Reference Genomics (Q-PHIRE Genomics), Forensic and Scientific Services, Queensland Department of Health, Coopers Plains, QLD Australia; 3 Victorian Infectious Diseases Reference Laboratory, Royal Melbourne Hospital at The Peter Doherty Institute for Infection and Immunity, Melbourne, VIC Australia; 4 Department of Infectious Diseases, University of Melbourne at The Peter Doherty Institute for Infection and Immunity, Melbourne, VIC Australia

**Keywords:** Infectious diseases, Policy and public health in microbiology, Comparative genomics, SARS-CoV-2, Viral epidemiology

## Abstract

The COVID-19 pandemic has driven demand for integrated genomics, resulting in fast-tracked development of AusTrakka, Australia’s pathogen genomics platform. This facilitated rapid data sharing, democratised access to computational and bioinformatic resources and expertise, and achieved national real-time genomic surveillance.

## Abstract (web summary)

The COVID-19 pandemic has accelerated the demand for near real-time analysis and dissemination of pathogen genomic data. In this comment, the authors describe how Australia has developed and rolled out its SARS-CoV-2 genomics platform, AusTrakka, and used it to support public health action.

The COVID-19 pandemic has highlighted the power of pathogen genomics to enhance infectious diseases surveillance and inform targeted public health responses^1^. Australia is a federation of states and territories, and public health laboratory activities have often been siloed between jurisdictional boundaries, hampering rapid genomic data sharing. This represents a major “missed opportunity” as pathogens do not respect borders and sharing of genomic information identifies and informs more rapid responses to infectious disease outbreaks^2,3^.

Recognising the significant opportunities afforded by pathogen genomics, there has been major government investments in the development and implementation of whole-genome sequencing (WGS), predominantly in high-income settings. The United Kingdom rapidly established the COVID-19 Genomics UK Consortium (COG-UK) and the United States government invested US$1.7 billion to expand genomics capacity for COVID-19^4^. In Australia, the pandemic also highlighted the need for an integrated public health genomics surveillance system.

## International examples of a genomics analysis platform

In 2010, the GenomeTrakr network, established by the US Food and Drug Administration, was the first large-scale experiment that facilitated real-time genomic data sharing directly between laboratories from foodborne pathogens on a national level^5^. Its successes in earlier detection of outbreaks demonstrated that genomic data sharing could inform public health interventions^5^.

In the United Kingdom, Public Health England (PHE) invested heavily in centralised sequencing capacity, launching the PHE Pathogen Genomics Service in 2014^6^. The national scope of activities covered by PHE allowed for national level integration of genomic and epidemiological data which meant challenges were around harmonisation of information systems and rapid reporting of data to referral laboratories.

Canada, like Australia, has a complex health system whereby each Canadian Province has its own public health laboratory. To facilitate real-time data sharing the Integrated Infectious Disease Analysis (IRIDA) project (https://irida.ca/) was developed. The IRIDA software operates independently for each public health laboratory with an interface to communicate between IRIDA database. The open-source nature of this platform enabled laboratories to establish genomic epidemiology capacity, however, the ability for laboratories to operate the software independently meant that there was no guarantee for real-time data sharing.

## Demand for national genomics surveillance in Australia

In 2015, the Communicable Diseases Genomics Network (cdgn.org.au), comprised of representatives from public health laboratories across all jurisdictions in Australia and New Zealand, was established with an overarching aim to implement pathogen genomics in public health. AusTrakka was first proposed by the CDGN in 2016 as a system that would facilitate consistency in genomic data sharing and reporting to improve public health surveillance and response. The AusTrakka platform was developed and deployed by the Microbiological Diagnostic Unit Public Health Laboratory and governed by the CDGN. This platform was built by public health laboratories, for public health laboratories, ensuring that the architecture and the bioinformatics pipelines and analysis protocols were created within a National Association of Testing Authorities (NATA)/International Organization for Standardization (ISO) accredited public health laboratory environment.

The COVID-19 pandemic was the catalyst for further development and deployment of AusTrakka in 2020, with key factors leading to fast-tracking including: (i) national funding for COVID-19 genomics sequencing and analysis through CDGN and AusTrakka; (ii) expert advocacy that led to Commonwealth recognition of the importance of real-time genomics data for surveillance; (iii) early demonstrations of the utility of genomics for COVID-19 from some jurisdictions - landmark events including the genomic identification of a hotel quarantine breach being the single source of a large second wave in Victoria, Australia, which catapulted genomics into the public domain;^1,7,8^ (iv) demand for investigation into ‘mystery cases’ detected despite restricted international travel, hotel quarantine policies and internal border closures; and (v) a strong desire for rapid detection and reporting of SARS-CoV-2 Variants of Concern in a nationally consistent way to inform public health control measures such as length of hotel quarantine and lockdowns. Subsequently, public and political demand for genomic investigation into essentially every case nationwide became an expectation that could only be met by rapid and open data sharing between health departments and public health laboratories. AusTrakka played a crucial role in facilitating genomic data sharing and providing national surveillance and ‘fine grain’ analyses, particularly into ‘mystery’ cases, revealing transmission within and between jurisdictions.

## AusTrakka: Australia’s national genomics platform

**Fig. 1:**
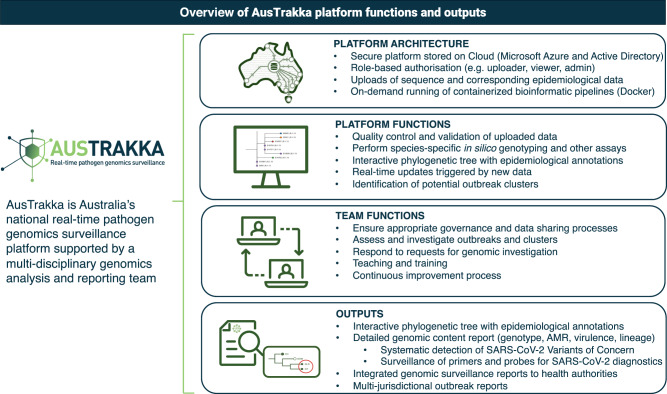
Overview of AusTrakka platform functions and outputs. The AusTrakka platform accepts sequence and epidemiological data from public health laboratories and performs several functions. Outlined in the figure is the platform architecture and functions, team functions and outputs of AusTrakka system.

AusTrakka is used by all public health laboratories in every jurisdiction in Australia and has extended to New Zealand. As public health laboratories upload new sequence data, the phylogenomic tree including all available sequences is updated. For SARS-CoV-2 a Maximum Likelihood tree is visualised, however the platform is able to visualise trees using any method. This tree is immediately accessible to public health laboratories to view their sequences in the national context. Given the sensitivity of this information, a specific framework for SARS-CoV-2 data sharing and analysis using AusTrakka was developed, formalising the endorsement from all jurisdictions to rapidly upload all SARS-CoV-2 sequence data to the AusTrakka platform. A multi-disciplinary and multi-jurisdictional AusTrakka team comprised of bioinformaticians, software engineers and genomic epidemiologists was established to support the development and operation (Fig. 1).

Public health laboratories had varying degrees of sequencing and bioinformatics capacity, however, through the deployment of AusTrakka it became clear that equitable access was important to ensure a comprehensive national dataset and reduce potential biases in the data. To facilitate this, the dedicated AusTrakka team provides analysis, interpretation, and reporting support to jurisdictions with limited capacity. Implementing this centralised and collaborative system ensured harmonisation of analyses, consistent national reporting, and provides all public health laboratories with the same computational resources and capability. At a national level, AusTrakka provides a focal point for integrated SARS-CoV-2 genomic surveillance information for public health agencies: outputs include reporting against genomics surveillance indicators as part of the Australian Government Communicable Diseases Intelligence Report, weekly reporting of Variants of Concern and investigation into multi-jurisdictional outbreaks (Fig. 1).

The AusTrakka development process revealed significant gaps and opportunities in the national public health system, reflecting Australia’s status as a federation. Public health in Australia is a complex multi-levelled system, with each jurisdiction largely responsible for their own public health service delivery, creating barriers to rapid genomic data sharing. Genomic data is generated by the public health laboratories and epidemiological metadata is collected by the health departments. Through the deployment of AusTrakka the significant benefits of integrated genomic data sharing were highlighted, however some limitations were also revealed, including the lack of interoperability between surveillance systems at all levels and the fact custodianship of genomic and epidemiological data is managed by separate systems.

A crucial element in the success of AusTrakka was its endorsement by, and compliance with, jurisdictional and federal governance structures for rapid data sharing between states and territories through a centralised platform. On 1 October 2020, AusTrakka was endorsed by the Australian Health Protection Principal Committee (AHPPC), the key decision-making committee for public health emergencies, however a key feature of this endorsement was ensuring access to the data was restricted to approved public health laboratories and agencies.

AusTrakka streamlines genomic and epidemiological data sharing and enables fine-grained access controls of data between public health laboratories. Within 12 months from endorsement, more than 33,000 high-quality SARS-CoV-2 sequences had been uploaded to AusTrakka, equal to approximately 50% of all COVID-19 cases in Australia, from over 80 users across all public health laboratories. AusTrakka has informed public health decision making throught demonstration of inter-jurisdictional transmissions resulting in internal border closures^1^, detection of Variants of Concern in real-time and in silico testing of primers and probes to identify target failures that may impact diagnostic tests (Fig. 1). The next stages of AusTrakka sees the real-time sharing and enhanced integration of epidemiological data on a national level and developing interoperability within existing national systems.

## Conclusion

AusTrakka has transformed the way multi-jurisdictional infectious diseases outbreaks are investigated and managed in Australia, demonstrating that barriers to genomics data sharing and centralisation could be overcome. As genomics capacity continues to develop globally, AusTrakka has provided a roadmap for integrating pathogen genomics into public health systems. There are, however, significant differences in global access to genomics technologies, though the principles of robust data governance, and the need for systems for rapid data sharing and analysis, and equitable access to computational capacity to enable real-time pathogen genomics surveillance, universally applies. So far, AusTrakka has been used as a core public health tool, although it has potential to be a valuable resource for advancing genomics research that will be explored in future.

It is imperative that the learnings and achievements experienced during the pandemic are sustained for the ongoing modernisation of infectious diseases surveillance and control. AusTrakka is adapting the approach to COVID-19 for other pathogens for broader public health impact, similar to that of the COG-UK, who have plans to forward the experience to the UK Health Security Agency to inform the development of a national pathogen genomics service. Sustained investment and engagement are critical to take full advantage of this enhanced genomics surveillance capability for other nationally notifiable pathogens that require close monitoring and targeted responses to reduce the public health, social and economic impacts of infectious diseases.

## Data Availability

No relevant data is available for this publication.

## Code availability

No relevant code is available for this publication.
